# Supplementary material for: High Glucose Treatment Limits Drosha Protein Expression and Alters AngiomiR Maturation in Microvascular Primary Endothelial Cells via an Mdm2-dependent Mechanism
Source: Cells. 2021 Mar 27;10(4):742. doi: 10.3390/cells10040742 (PMC8065922; doi:10.3390/cells10040742)
Supplement: Supplementary file 1 [file cells-10-00742-s001.pdf]

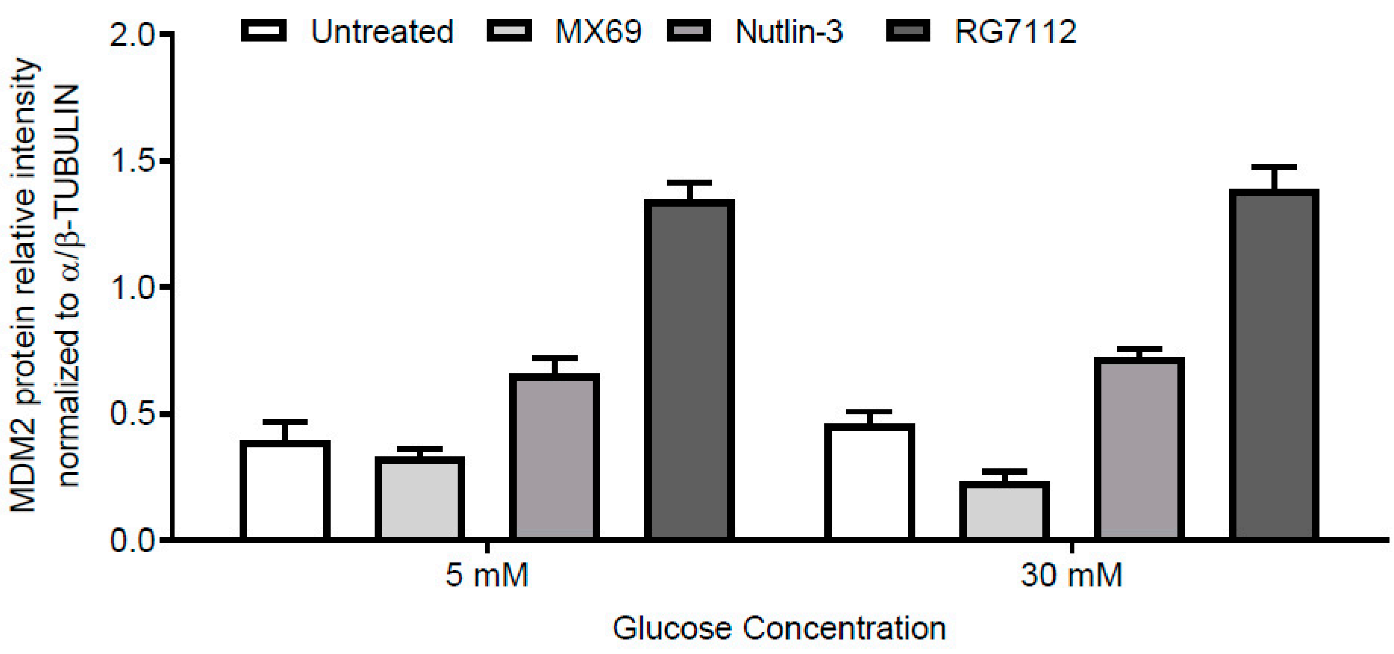

Supplementary Figure S1. Human dermal microvascular endothelial cells (HDMECs) were treated with 30 mM or 5 mM glucose and Murine Double Minute-2 (MDM2) inhibitors (MX69, Nutlin-3 and RG7112) for 6 h. Levels of MDM2 protein were measured by western blot. α,β-TUBULIN was used as a loading control. Quantification shows means ± SEM, $n = 6$. Two-way ANOVA shows an overall effect of MDM2 activity inhibition, $p < 0.0001$. Nutlin-3 and RG7112 treated cells presented significant higher level of MDM2 than untreated cells. MX69 treated cells showed a lower level of MDM2 than un-treated cells.

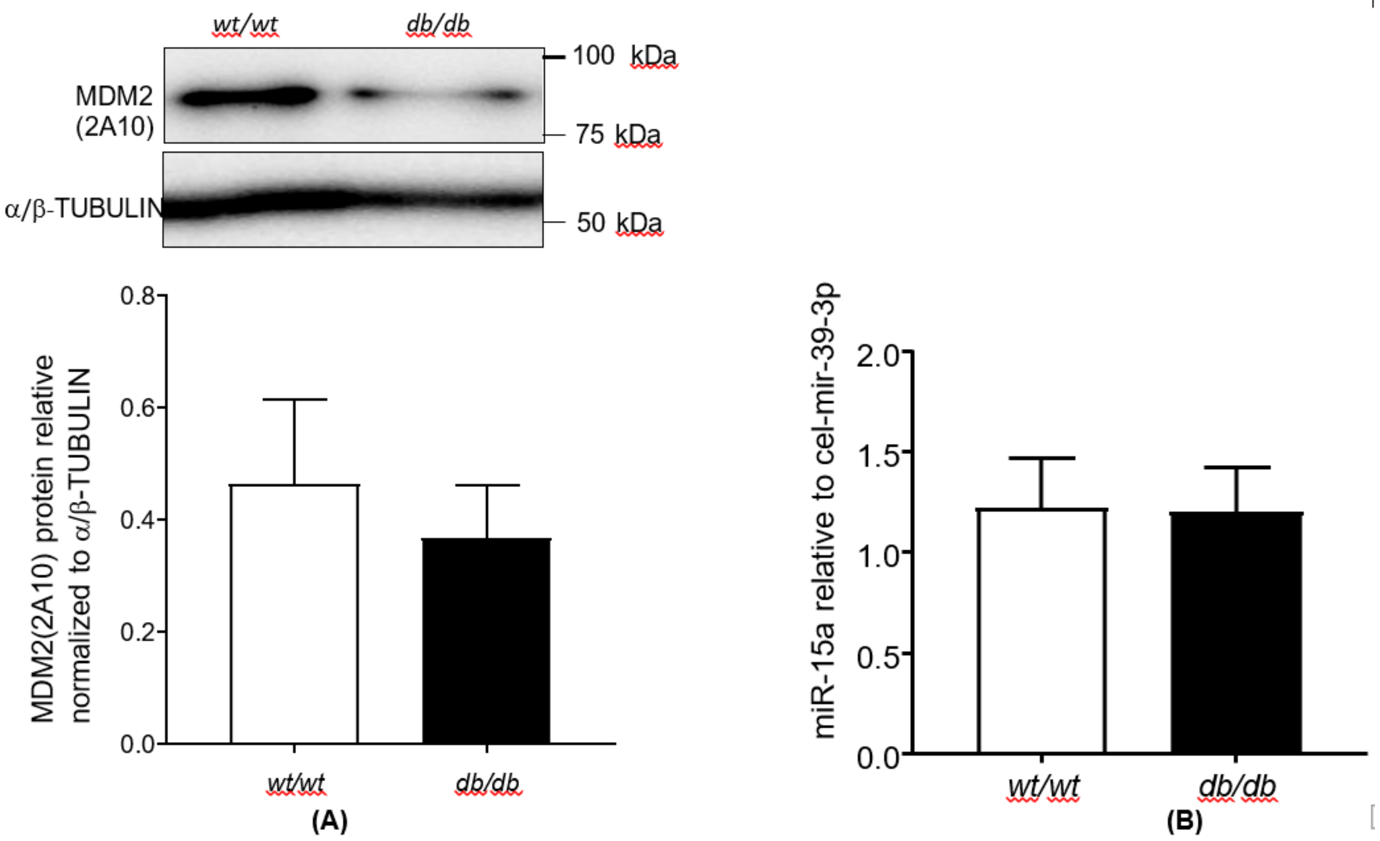

Supplementary Figure S2. Level of MDM2 protein and miR-15a are similar in the gastrocnemius skeletal muscle of wild type and *db/db* mice aged 13 weeks. (**A**) MDM2 protein levels in *wt/wt* and *db/db* mice as measured by 2A10 antibody. α,β-TUBULIN was used as a loading control. (**B**) Mature miR-15a level relative to exogenous spike-in cel-miR-39-3p. Data are means ± SEM (*wt/wt* mice n = 8 and *db/db* mice n = 6).
